# First 3 Minutes: A Rapid Cycle Deliberate Practice Pediatric Resuscitation Simulation for Multidisciplinary Staff

**DOI:** 10.15766/mep_2374-8265.11529

**Published:** 2025-06-06

**Authors:** Kathryn Songer, Marie Fiero, Joan Roberts

**Affiliations:** 1 Third-Year Fellow Physician, Division of Pediatric Critical Care Medicine, Spencer Fox Eccles School of Medicine at the University of Utah and Primary Children's Hospital; 2 Assistant Professor, Division of Pediatric Critical Care Medicine, University of Washington School of Medicine and Seattle Children's Hospital; 3 Professor, Division of Pediatric Critical Care Medicine, University of Washington School of Medicine and Seattle Children's Hospital

**Keywords:** Simulation, Pediatrics, Interdisciplinary Medicine, CPR, Resuscitation, Code Blue, Cardiopulmonary Resuscitation, Rapid Cycle Deliberate Practice, Critical Care Medicine

## Abstract

**Introduction:**

Medical staff on acute care wards respond to in-hospital emergencies, yet they are often poorly prepared due to the infrequency of pediatric cardiopulmonary events. We developed this rapid cycle deliberate practice simulation to improve the skills and knowledge required to initiate high-quality CPR and prepare for the arrival of the code blue response team.

**Methods:**

This 30-minute simulation was performed in situ within hospital rooms and required low-fidelity equipment. Participants resuscitated an unresponsive, pulseless infant. The simulation was conducted three times, with each participant cycling through each role. Critical actions included checking for responsiveness and calling for help, starting compressions, ventilating the patient, and placing defibrillation pads while maintaining high-quality CPR.

**Results:**

Participants' (attending physicians', pediatric trainees', advanced practice providers', registered nurses') self-reported confidence, as rated based on a 5-point Likert scale (1 = *strongly disagree*, 5 = *strongly agree*), significantly improved in providing high-quality CPR (*n* = 151; *Z* = 9.4; *p* < .01) and managing airway, breathing, and circulation in the first 3 minutes of a code situation (*n* = 154; *Z* = 9.6; *p* < .01). Knowledge scores (four multiple-choice questions) assessing high-quality CPR principles improved from a mean 68% to 85% (*n* = 151; *p* < .01). Respondents indicated they found the training session to be helpful, with a mean score of 4.8 on a 5-point Likert scale (1 = *not at all helpful*, 5 = *extremely helpful*).

**Discussion:**

This multidisciplinary simulation was well received and improved participants' confidence in responding to pediatric cardiopulmonary emergencies and knowledge of high-quality CPR principles.

## Educational Objectives

By the end of this activity, learners will be able to:

1. Identify an unresponsive patient and call for help appropriately.
2. Increase confidence in addressing airway, breathing, and circulation in the first 3 minutes of a code situation.
3. Recall high-quality cardiopulmonary resuscitation principles.

## Introduction

Acute care floor medical staff are often the first responders to in-hospital pediatric cardiopulmonary emergencies prior to the arrival of the code blue response team. Acute care staff, including bedside nurses, respiratory therapists, advanced practice providers, and medical trainees, typically receive training in responding to medical emergencies every 2 years in the form of certification in pediatric advanced life support (PALS), basic life support (BLS), or pediatric emergency assessment, recognition, and stabilization (PEARS). Despite this training, acute care staff are often poorly prepared to be the first responders to in-hospital medical emergencies due to the infrequent, yet critical, nature of true pediatric cardiopulmonary events, with only 7% of in-hospital pediatric cardiopulmonary resuscitation (CPR) provided on general wards.^1^ It has been shown that resuscitation knowledge and clinical skills performance diminish over time.^2^ Without the opportunity to combat knowledge and skill loss, there is a risk for suboptimal resuscitation outcomes for patients who decompensate on the pediatric ward. Additionally, there is potential for health care staff to experience trauma if they feel inadequately prepared to respond to code events. Simulation has been suggested as a methodology to reinforce resuscitation skills and knowledge.

This simulation aimed to provide brief, focused training to increase competency and confidence for acute care providers as first responders to in-hospital respiratory and cardiac emergencies. This simulation focuses on the hands-on, practical skills required to initiate CPR and prepare for the arrival of the code blue team. As opposed to the typical debriefing and reflective learning experienced in many simulations, this simulation uses rapid cycle deliberate practice (RCDP), a technique that involves learners repeatedly performing the same simulation with micro-debriefs interjected by the instructor.^3,4^ RCDP in the pediatric medical training setting has been well received and effective in improving resuscitation performance and participant confidence.^5–7^ This is the first known simulation aimed at increasing the confidence and competence of multidisciplinary acute care staff in responding to code situations using RCDP techniques.

## Methods

### Development

We developed this simulation after debriefs for multiple code events on the acute care floor revealed that pediatric residents and other bedside caregivers did not feel adequately prepared as first responders. We met with pediatric residents and other acute care staff to elicit ideas for a simulation training that would address this deficit. Using this information, we constructed a simulation scenario aimed at improving first responders' confidence and competence in a code situation. We developed the materials to allow multiple facilitators to have a standardized approach to facilitating the sessions. The University of Washington Institutional Review Board determined this study to be exempt on September 2, 2021 (No. 00013934).

The simulation was performed in situ in hospital rooms or infusion clinic treatment rooms at Seattle Children's Hospital as part of a voluntary simulation curriculum. Facilitators were physicians within the critical care department (hospitalist, fellow, or attending). Facilitators were required to have participated in at least one simulation and to have co-facilitated a simulation session prior to facilitating independently. Learners included attending physicians, pediatric residents, advanced practice providers, and registered nurses, who had previously completed training in PALS, PEARS, or BLS as a requirement for employment.

We asked the learners to complete an electronic questionnaire before the simulation and after debriefing. The three authors developed the questionnaire and it has not been validated. The questionnaire included two questions inquiring about learner's confidence in providing high-quality CPR, managing an airway, and responding to a code situation. The same questions were used pre- and postparticipation. Confidence was rated based on a 5-point Likert scale (1 = *strongly disagree*, 2 = *disagree*, 3 = *neither agree nor disagree*, 4 = *agree*, 5 = *strongly agree*). The questionnaire also included four multiple-choice questions assessing participants' knowledge of high-quality CPR principles, as well as some open-ended questions. On the postparticipation questionnaire, participants were asked “How helpful did you find this training session?” based on a 5-point Likert scale (1 = *not at all helpful*, 2 = *not so helpful*, 3 = *somewhat helpful*, 4 = *very helpful*, 5 = *extremely helpful*).

Because the data distribution is nonnormal with a Likert scale, Wilcoxon signed-rank test was used to compare confidence scores before and after the simulation. For the multiple-choice test results, a one-tailed paired *t* test was used to compare knowledge scores before and after the simulation. A *p* value less than .05 was considered significant for all analyses.

### Equipment/Environment

We recommend the following equipment:

• Low-tech CPR-compatible mannequin (high tech could also be used if resources are available)
• Training code cart with backboard, defibrillator, and defibrillator pads
• Pediatric mask with inflatable cushion
• Pediatric self-inflating ventilation bag
• Stool (for compressions)

The simulation is optimally performed in a clinical care setting (e.g., acute care room). Other settings can be used, such as a conference room, but utilizing an authentic patient care room allows instructors to point out specific aspects that are important for code situations in the participant's clinical milieu.

### Personnel

The simulation facilitator was a critical care hospitalist, fellow, or attending who provided information regarding the patient's indication for hospitalization and physical examination findings. Other staff members, such as advanced practice providers, with expertise in responding to code situations could be facilitators.

### Implementation

We trained facilitators by having them first participate in a simulation session led by the three authors (Kathryn Songer, Marie Fiero, Joan Roberts), of whom one was a critical care hospitalist and the others are attending physicians in the critical care division. Next, the training facilitators cofacilitated a session with an individual who had been previously trained. A facilitator packet, which included a facilitator guide (Appendix A), the simulation scenario with critical action points (Appendix B), and facilitator scripts with teaching points (Appendix C), was shared in advance. In each simulation, the learners were multidisciplinary staff who may respond to a code situation on the hospital ward, including registered nurses, attending physicians, advanced practice providers, and pediatric trainees (medical students, residents [PGY levels 1–3], and fellows [PGY levels 4–6]). We offered this simulation to potential participants primarily through email invitations from nursing education leaders and chief medical residents and by inviting trainees on their pediatric critical care rotation. Leadership from acute care units had the opportunity to schedule simulation sessions for their employees.

The simulation was optimally designed for a group of three learners per session. More learners may participate through observation and cycling through the scenario, but it was primarily set up for three participants. On the day of the simulation, the facilitator identified an open patient care room for use and prepared by placing the mannequin on the bed, the pediatric mask and bag in/on the emergency airway box, and the training code cart outside the room. The facilitator printed the visual aid with simulation objectives (Appendix D) and taped it at the front of the room. The participant role cards were also printed by the facilitator to provide to the learners during the simulation (Appendix E).

The simulation sessions lasted approximately 30 minutes, with a pre- and postparticipation survey for each learner not included in that time frame. As learners arrived, the facilitator distributed the preparticipation survey using an electronic QR code (Appendix F). The facilitator started the session with the presimulation briefing and review of high-quality CPR components. The same scenario was then repeated three times after the facilitator assigned participant roles. The first simulation was performed in its entirety without any stoppages and was followed by a debrief and teaching session using the provided teaching points. The simulation case was then repeated two more times, with each participant cycling through each role. The simulation was stopped by the facilitator and repeated in the second and third repetitions if any of the critical action points were omitted by participants as suggested in the simulation scenario. The facilitator instructed the learners to complete a postparticipation survey (Appendix G) at the end of the simulation session and distributed a key take-home points sheet (Appendix H).

### Approximate Timing

Prior to the official start of the session, participants were asked to complete a preparticipation survey. The session began with a 5-minute segment that includes introductions, an orientation to the simulation environment, and a brief review of high-quality CPR principles. This was followed by the first simulation, which took place from minutes 5 to 10. The next 10 minutes (minutes 10–20) were dedicated to focused instruction on calling for help, performing basic airway skills, and managing the timing and choreography of defibrillator pad placement. From minutes 20 to 28, participants engaged in two additional simulations, incorporating lessons on optimizing the physical and team environment. The final 2 minutes (minutes 28–30) were reserved for a debrief and distribution of key take-home points. After the session concludes, participants completed a postparticipation survey.

### Debriefing

Debriefing was focused on the preidentified teaching points and practical skills. After the first simulation, the facilitator asked one or two brief questions, such as “What was hard about that?” The facilitator did not spend too much time in reflection but ensured that they answered any specific questions that came up during the simulation. Three mini-lectures from the facilitator script (Appendix C) were typically given after the first simulation. The fourth mini-lecture was typically given after the second or third simulations. Following the final debrief, learners immediately completed a postparticipation survey.

### Assessment

After the first simulation, the facilitator completed the critical actions checklist (Appendix B). Learners received feedback following the case during the debriefing and mini-lessons. During the second and third simulations, learners were stopped in the middle of the case and provided with feedback if a critical action step was missed. After feedback, the case was restarted from the beginning or at a point just before stopping to give more opportunities to practice these skills. Additionally, the pre- and postparticipation questionnaire included four multiple-choice questions aimed at assessing knowledge of high-quality CPR components. Answers were compared pre- and postparticipation to measure the effectiveness of the simulation. The questionnaire inquired about learners' takeaways and suggestions for improvement.

## Results

Between September 2021 and August 2024, 229 participants completed the preparticipation survey, and 189 participants completed the postparticipation survey. Paired responses were completed for 154 participants, and only the first time participating was included if a learner participated in more than one session. The analyses were conducted on the paired surveys. Additional survey elements were added after the first pilot of the session, and therefore some survey elements had only 151 responses, which are denoted in each table. The 154 participants who completed a pre- and postparticipation survey included 71 advanced practice providers, 44 resident/fellow physicians, 29 nurses, six attending physicians, and four medical students. See Table 1 for participants' roles and number of years in that role.

**Table 1.**
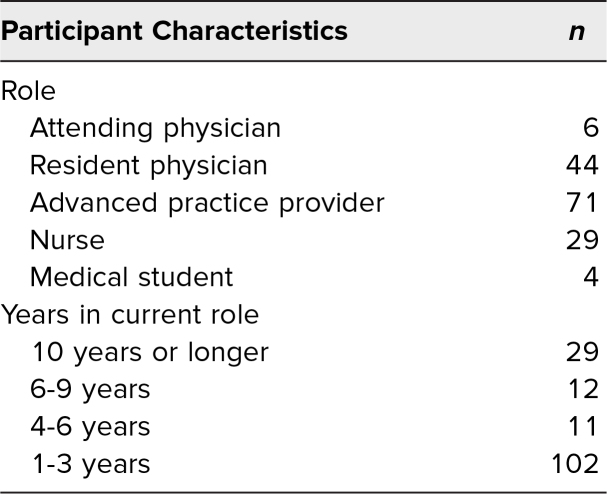
Participant Demographics and Years of Experience (*N* = 154)

We evaluated participant's self-reported rankings of confidence in responding to code events pre- and postparticipation. As shown in Table 2 and the Figure, the self-reported response scores to the Likert-scale items (1 = *strongly disagree*, 5 = *strongly agree*) indicated an increase in confidence in each category among the overall cohort and among participants stratified by role (advanced practice providers, resident/fellow, and nurse). There were not enough participants in the attending physician and medical student groups to run these analyses with adequate power. While each participant's acquisition of skills was not formally documented, the process of RCDP allowed for the facilitator to stop each simulation and have participants demonstrate the critical skills (e.g., calling for help, ventilation with bag device, high-quality CPR, room optimization), if they were not being performed adequately.

**Table 2.**
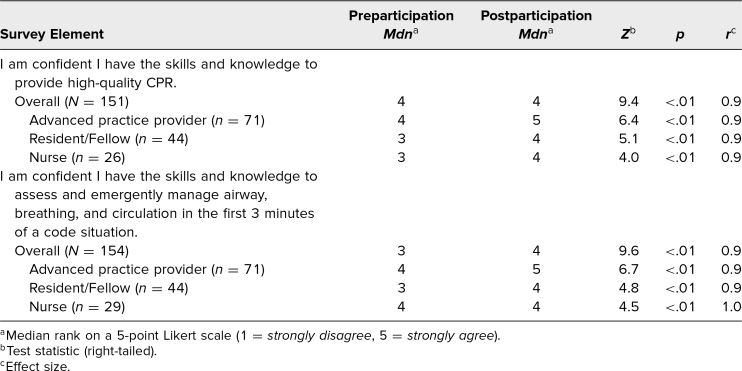
Participant Self-Assessed Confidence Pre- and Postparticipation Compared Using Wilcoxon Signed-Rank Test

**Figure.**
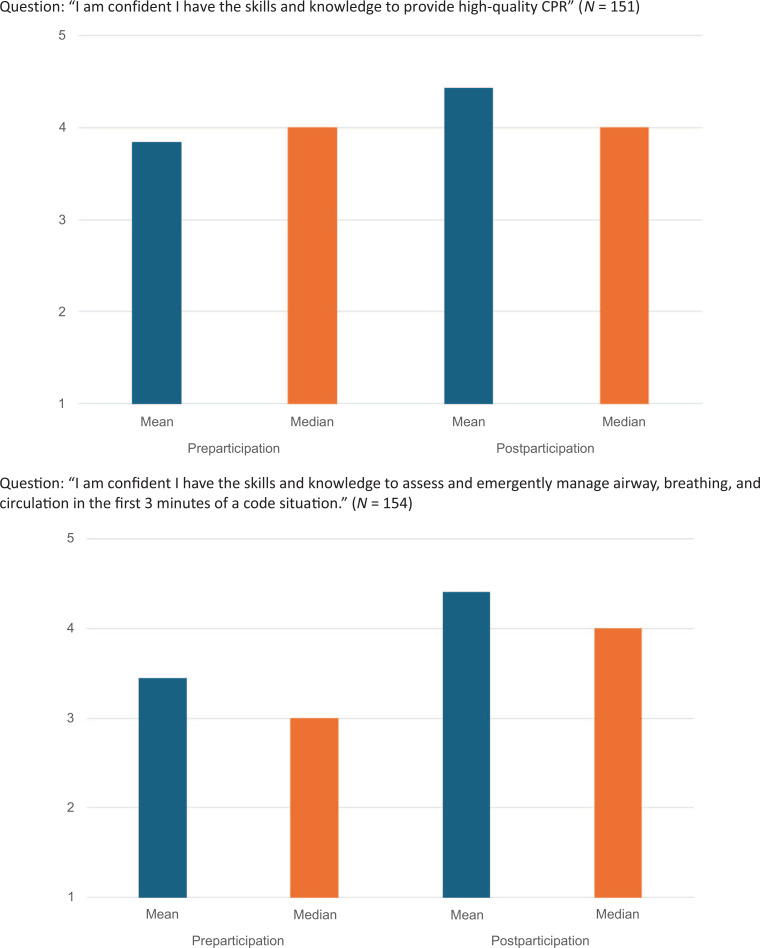
Mean and median self-assessed confidence scores for the overall cohort before and after participation in the pediatric CPR simulation. Rank is based on a 5-point Likert scale (*1 = strongly disagree, 5 = strongly agree*).

Participants were also tested pre- and postparticipation on knowledge of principles of high-quality CPR, using four multiple-choice questions. Scores for the correct answer on these multiple-choice tests improved after the simulation session, with an overall mean score of 68% preparticipation and 85% postparticipation (*p* < .01; Table 3). When stratified by participant role, the advanced practice providers and resident/fellow groups showed significantly improved knowledge scores; however, improvement in scores in the nurse group did not reach statistical significance despite having pre- and postparticipation scores similar to those of the other groups, likely due to the smaller sample size in this group. There were not enough participants in the attending physician and medical student groups to run these analyses with adequate power.

**Table 3.**
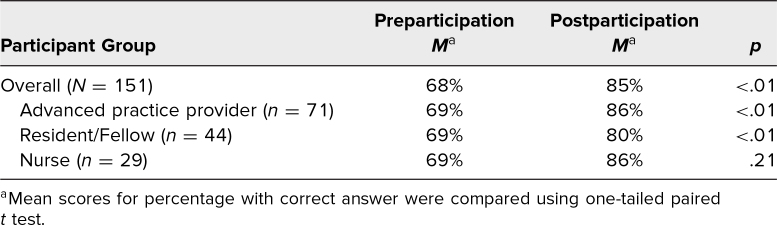
Knowledge Scores of High-Quality CPR Principles on Pre- and Postparticipation Multiple-Choice Tests

Postparticipation, participants were asked “How helpful did you find this training session?” The majority of participant responses indicated that they rated the session as very or extremely helpful, with a mean score of 4.8 (*n* = 154) on 5-point Likert scale.

Participants were encouraged to provide comments regarding a learning takeaway and asked for any suggestions for improvement. Representative main takeaways from the surveys highlighted several key topics. Many emphasized the importance of clear communication during resuscitation events, noting the need to verbalize actions and speak up to support team coordination and role optimization. One participant succinctly stated, “Communication is key! Limit CPR interruptions!” Others appreciated the practical orientation to the physical environment, with comments focusing on learning how to use the equipment in the room effectively and adapting to varied room setups and resources. Flexibility and situational awareness were also noted as valuable, especially the reminder that hospital environments could differ significantly, with the comment that there were “many different room setups, beds, etc.” Participants also found it particularly helpful to be introduced to hospital-specific equipment and expressed appreciation for the approach of pausing simulations to address and correct techniques in real time. Lastly, learners recognized the importance of early action, reflecting on how they could contribute meaningfully before the code team arrived by initiating CPR and calling for help. The most common feedback received was that this training should occur more frequently and in different locations throughout the hospital to experience the variation in specific rooms.

## Discussion

Overall, we report on a successful and unique multidisciplinary simulation training program that was enthusiastically received and effective in improving participant confidence in responding to pediatric cardiopulmonary emergencies and in improving participants' knowledge of high-quality CPR principles. The strengths of this simulation are the brief, focused nature and simple objectives that allow for generalizability across medical disciplines and settings. The curriculum builds upon the knowledge that participants have obtained in previous courses (i.e., PALS, PEARS, etc.) and allows for the practice of life-saving skills with the guidance of an experienced facilitator. The RCDP method was chosen to allow participants to master basic skills before progressing to the next step of the resuscitation. A 2019 qualitative evaluation of learners' experiences during RCDP simulations found that RCDP increases participant confidence in code scenarios by introducing new information in smaller chunks and reinforcing learning in real time.^8^ This correlates well with the qualitative feedback received from our learners after this simulation and the significant increase in confidence scores postparticipation. Other simulation studies have shown that participants in RCDP simulations had improved resuscitation performance in terms of time to pulse check or defibrillation and reported lower stress levels associated with their experience compared to standard postevent debriefing.^9–11^ These are ideal advantages for a brief, skill-focused simulation. Future studies will be geared toward quantitatively evaluating code metrics during and after participation in this simulation.

The development of this curriculum was not without challenges. The earliest versions of this simulation revealed core issues that prevented the simulation session from being conducted efficiently. For example, we found that learners were so unfamiliar with resuscitation equipment that pilot simulations were derailed by even the act of locating supplies within the hospital room. We modified the simulation sessions to include a more extensive orientation of the room, including the location of the code button, defibrillator/code cart, and airway equipment. The orientation was also modified to demonstrate attaching pads to the defibrillator. Additionally, learners were unfamiliar with airway and ventilation techniques, so a specific mini-lecture to cover this information was delivered between simulations. The simulation sessions became more efficient and adhered to the ideal time line once we added a more extensive orientation to the equipment.

There are some limitations to this simulation intervention. Postparticipation surveys demonstrated improvement in participant confidence and knowledge, but it is only reflective of a single time point immediately after the training session. We did not assess participant retention of these skills or knowledge at a later time. The desire for a higher frequency of this type of training was common in the participant feedback. Further work will consider the limitations inherent to scheduling educational sessions with different types of learners with variable schedules. This curriculum has been integrated into our pediatric intensive care unit rotation for pediatric residents, allowing us to reach many learners. We have been overall pleased with our ability to deliver this content to multidisciplinary staff, but we realize it could be difficult to schedule or implement a similar curriculum at other institutions, depending on staffing structures and educational practices. Additionally, the pre- and postparticipation questionnaires have not been validated. The high-quality CPR knowledge test had one question that included verbiage using “not,” which may have impacted testing as learners performed most poorly on this question. If we throw this question out (question #4), the pretest mean score is 75%, which improves posttest to a mean score of 95% (*p* < .01). Regardless, our results show, with or without that particular question, that knowledge of high-quality CPR parameters improved after the intervention.

### Future Directions

Ideally, each staff member who works on the acute care floor and who could be a first responder to a code should be able to participate in this simulation multiple times per year to avoid skills loss. While we have made great strides in reaching different staff members, we have not yet been able to create a program structure that will ensure all employees are reached. Ultimately, the goal would be to determine if resuscitation outcomes improve over time after implementing this simulation curriculum.

## Appendices


First 3 Minutes Facilitator Guide.docxSimulation Scenario with Critical Action Points.docxFacilitator Scripts and Teaching Points.docxVisual Aid with Simulation Objectives.docxPrintable Team Role Cards.docxPreparticipation Survey and CPR Test.docxPostparticipation Survey and CPR Test.docxKey Take-Home Points for Learners.docx
*All appendices are peer reviewed as integral parts of the Original Publication.*
