# Supplementary material for: First 3 Minutes: A Rapid Cycle Deliberate Practice Pediatric Resuscitation Simulation for Multidisciplinary Staff
Source: MedEdPORTAL. 2025 Jun 6;21:11529. doi: 10.15766/mep_2374-8265.11529 (PMC12141546; doi:10.15766/mep_2374-8265.11529)
Supplement: Supplementary file 1 — First 3 Minutes Facilitator Guide.docxSimulation Scenario with Critical Action Points.docxFacilitator Scripts and Teaching Points.docxVisual Aid with Simulation Objectives.docxPrintable Team Role Cards.docxPreparticipation Survey and CPR Test.docxPostparticipation Survey and CPR Test.docxKey Take-Home Points for Learners.docx [file mep_2374-8265.11529-s001.zip › B. Simulation Scenario with Critical Action Points.docx]

| **Appendix B: Simulation Scenario and Critical Action Points**  **SIMULATION CASE TITLE: First 3 Minutes Simulation**  **AUTHORS: Kathryn Songer, Marie Fiero, Joan Roberts**  **LEARNER AUDIENCE: Multidisciplinary pediatric medical staff** | |
| --- | --- |
| **PATIENT NAME: Susie**  **PATIENT AGE: 6 months**  **CHIEF COMPLAINT: Unresponsive**  **PHYSICAL SETTING: Pediatric hospital ward room** | |
| **Brief Narrative Description of Case** | A 6-month old girl admitted for bronchiolitis is noted to be unresponsive by a parent |
| **Primary Learning Objectives** | 1. Identify an unresponsive patient and call for help appropriately 2. Increase confidence in addressing airway, breathing, and circulation in the first 3 minutes of a code situation 3. Recall high-quality CPR principles |
| **Critical Actions** | 1. Check for responsiveness and call for help when child is unresponsive 2. Check for a pulse, start high-quality compressions if no pulse 3. Open airway and begin ventilation with 15:2 ratio with compressions 4. Roll patient, place backboard and defibrillation pads |
| **Learner Preparation or Prework** | Learners will be oriented to the simulation environment and equipment. Facilitator will review high-quality CPR principles prior to simulation. |

| Initial Presentation | | | |
| --- | --- | --- | --- |
| **Initial Vital Signs** | HR: 20, BP: 0/0, RR: 0, O2 Sat: not reading, T: 37 C, Weight: 8 kg – don’t offer verbally, patient is not on monitors | | |
| **Overall Setting and Appearance** | Hospital Ward Room  Infant in crib  Sign on bed with weight of patient and code drug doses as usual for admitted patients.  Cyanotic. No cough, no crying. | | |
| **Standardized Participants (and Their Roles in the Room at Case Start)** | Parent (voiced by facilitator): Role is to provide information below | | |
| **HPI** | Initial call for help: “Help, my daughter needs help! She’s stopped responding!”  If asked why she was admitted: “She was admitted for bronchiolitis.” | | |
| **Past Medical/Surgical History** | **Medications** | **Allergies** | **Family History** |
| None | Oxygen | None | None |
| **Physical Examination:** (if not filled in, normal) | | | |
| **General** | Limp, no crying | | |
| **Lungs** | No breath sounds initially, crackles bilaterally when ventilated | | |
| **Cardiovascular** | Pulseless, no heart tones initially | | |
| **Neurological** | Unresponsive | | |
| **Skin** | Cool to touch, mottled appearance, delayed cap refill | | |

| Instructor Notes - Changes and CASE Branch Points | | |
| --- | --- | --- |
| **Intervention / Time Point** | **Change in Case** | **Additional Information** |
| *When the parent (facilitator) calls for help, the first responder can enter the room immediately* |  |  |
| *First responder calls for help or hits code button* | *At this time, second responder will come in immediately while the remaining team members will go and retrieve the code cart* |  |
| *15-30 seconds after the second responder enters, the third responder can bring in the code cart* |  | *Goal is to mimic the normal delay of getting the code cart* |
| *STOP simulation when team has demonstrated high-quality CPR and appropriate ventilation technique with the defibrillation pads in place* |  |  |

**Ideal Scenario Flow**

Case started by parent role: “Help, my child needs help! She stopped responding!”

First Responder: Enters the room, checks for responsiveness, sees no response. Shakes and shouts to make sure. [Baby should be quiet]

“Patient is unresponsive. I’m calling for help!” Yells for help or hit code button if nearby.

Expected Actions:
First Responder conducts rapid assessment of perfusion, respiratory examination including airway, breath sounds.

- First responder feels for central pulse 5-10 seconds
- Pulses should not be found, says “There is no pulse, I’m starting compressions.” and starts compressions

(Stop simulation if not delivering high quality CPR)

Second Responder: Enters the room and sees compressions ongoing.

- Ask: “Has a code been called?” If not, call code.
- Second responder becomes *airway*, helps position bed/patient, gets BVM, and starts coordinated CPR (15:2).

(Stop simulation if airway responder is not well positioned to attend to airway and ventilation or if not using equipment appropriately)

Third responder: Arrives w/ code cart. Takes charge and optimizes resuscitation measures.

- Say: “Continue compressions. I will get the pads ready.”
- Coordinate flip with end of round of compressions. “On my count, turn patient and I will place back board and back pad.”

Other actions: Place stool for compressor. Optimize environment to prepare for arrival of code team.

STOP simulation when team has demonstrated high-quality CPR and appropriate ventilation technique with the defibrillation pads in place. Repeat two more times with learners rotating roles.

**Anticipated Management Mistakes**

*The goal of these RCDP simulations is to gain skill competency and confidence. As such, we modified the simulation session to include orientation and pre-briefing materials that would allow them to focus on skill acquisition over knowledge base.*

1. *Confusion regarding roles: Since this is a multidisciplinary simulation, learners might not know which role to take. We want each learner to practice each role, as each staff member could potentially respond to a code event in a different sequence. To take away the confusion regarding typical roles (such as who might naturally lean towards airway, code leader, etc.), we pre-assign roles. We remind learners that establishing clear roles in a critical situation is especially important to prevent errors of omission. For each simulation, we decide in advance who would be 1st, 2nd, and 3rd responder. We also provide “role cards” to learners to remind them of each responder’s role. It is possible that participants feel uncomfortable in these roles, but they should be reminded that they should be able to perform all of these skills in an emergency.*
2. *Forgetting to check for pulse: Since the same scenario is repeated multiple times, learners sometimes skip the step of assessing the patient initially. The 2nd and 3rd simulations should be stopped if the first responder does not assess the patient appropriately, and the learners should be allowed to start again.*
3. *Poor CPR technique: Many of our learners had never performed CPR outside of PALS or PEARS courses. We want learners to be familiar with CPR prior to the start of the simulation, so we include a review of high-quality CPR principles in the pre-simulation orientation. Learners frequently have not practiced high-quality CPR technique, and the 2^nd^ and 3^rd^ simulations should be stopped if CPR technique is not adequate. Learners should receive feedback on how to improve compressions and then be allowed to try again.*
4. *Unfamiliarity with resuscitation equipment: Learners frequently do not know where to find resuscitation equipment or how to use it. We modified the simulation pre-brief to include an orientation to the room, including the location of the code button, defibrillator/code cart, and airway equipment; however, learners may still not use equipment properly. A specific mini-lecture to cover this information is delivered between simulations, and the 2^nd^ and 3^rd^ simulation should be stopped if equipment is not being used properly. Learners should receive feedback on how to improve and then be allowed to try again.*
