# Supplementary material for: First 3 Minutes: A Rapid Cycle Deliberate Practice Pediatric Resuscitation Simulation for Multidisciplinary Staff
Source: MedEdPORTAL. 2025 Jun 6;21:11529. doi: 10.15766/mep_2374-8265.11529 (PMC12141546; doi:10.15766/mep_2374-8265.11529)
Supplement: Supplementary file 1 — First 3 Minutes Facilitator Guide.docxSimulation Scenario with Critical Action Points.docxFacilitator Scripts and Teaching Points.docxVisual Aid with Simulation Objectives.docxPrintable Team Role Cards.docxPreparticipation Survey and CPR Test.docxPostparticipation Survey and CPR Test.docxKey Take-Home Points for Learners.docx [file mep_2374-8265.11529-s001.zip › C. Facilitator Scripts and Teaching Points.docx]

Appendix C: Facilitator Scripts and Teaching Points

This appendix includes scripting for the Pre-Simulation Brief and Teaching Points to be used during the simulation session.

Scripting for Pre-Simulation Briefing

After introducing yourself as facilitator and having the participants introduce themselves, read and discuss each of the following:

**1. Safe Learning Environment:**

**Basic Assumption**: All of us are well-trained, compassionate people who came to do the best we can do and learn along the way. All of you have received the training necessary to complete today’s activities, but it is expected that you will make mistakes, as most people have not received enough practice with these skills to perform them perfectly. That’s why we are here today -- to practice in a safe space.

**Confidentiality:** If you learn something new today about how to care for patients, please share with others; however, don’t share the details of the actions (good or bad) of your colleagues. No one is being evaluated, so help each other as you would during an actual resuscitation.

**Fiction Contract:** There are parts of today’s simulation that are not completely realistic, but try to act as you would in a real scenario.

**2. Orientation to Simulation:**

**Simulation location:** You and your colleagues are rounding on patients, when you hear a call for help from a parent in a room with a patient that is not on your service.

**Roles:** Establishing clear roles in a critical situation is especially important to prevent errors of omission. For example, if there is a specific person in the respiratory role even when the patient is breathing spontaneously, it is less likely that apneic spells will be missed. *(Distribute role cards)*

For each simulation, we will decide in advance who will be 1^st^, 2^nd^, and 3^rd^ responder. The first responder should focus on initial rapid assessment, calling for help, and then turning back to pulse and perfusion. The second responder should focus on airway, oxygen, ventilation. The third responder should focus on applying defibrillation pads and placing backboard if needed.

When the parent calls for help, the first responder can enter the room immediately. Start your assessment and call for extra help when needed. At this time, one of your colleagues will come in immediately while the remaining team members will go and retrieve the code cart. Before getting the code-cart and sliding it in the room, please wait 15 seconds to mimic the normal delay of getting the code cart.

**Orientation to room and mannequin:**

1) Mannequin - Please take all necessary actions to save the patient. Do not pretend to take actions, actually attach pads and do compressions. You may get some history from the parent in the room, but do not feel the need to comfort the parent; focus on the patient.

2) Code button - When you would hit the code button, just vocalize that you would do so.

3) Defibrillator equipment – This is a hospital defibrillator with training pads. The patient in this scenario is a child, so you would use the pediatric pads. (*Show learners how to attach pads and plug into defibrillator)*

4) Airway equipment – On acute care floors, emergency airway supplies are located in the clear box on the head wall of the room. There is a suction catheter and various sizes of masks and bags.

**Review of high quality CPR principles:**

With your learners, discuss the principles of high quality CPR (based on 2020 AHA guidelines^1^). Depending on learners’ level of confidence with CPR, either teach didactically or in an interactive question/answer format.

- providing adequate chest compression rate (≈100–120/min)
- providing adequate chest compression depth (depress the chest at least one third the anterior-posterior diameter of the chest, approximately 1.5 inches (4 cm) in infants to 2 inches (5 cm) in children.
- minimizing interruptions in CPR
- allowing full chest recoil between compressions
- avoiding excessive ventilation
  - In 2-rescuer CPR without an advanced airway, provide a compression-to-ventilation ratio of 15:2

**Rapid Cycle Deliberate Practice:** After the first run-through, simulations may be stopped in the middle of the action and you will be provided with feedback. After feedback, I will restart the case from the beginning or just before we stopped to give feedback to give you more opportunities to practice these skills.

Reference:

1. Topjian AA, Raymond TT, Atkins D, Chan M, Duff JP, Joyner BL Jr, Lasa JJ, Lavonas EJ, Levy A, Mahgoub M, Meckler GD, Roberts KE, Sutton RM, Schexnayder SM; Pediatric Basic and Advanced Life Support Collaborators. Part 4: Pediatric Basic and Advanced Life Support: 2020 American Heart Association Guidelines for Cardiopulmonary Resuscitation and Emergency Cardiovascular Care. Circulation. 2020 Oct 20;142(16_suppl_2):S469-S523. doi: 10.1161/CIR.0000000000000901. Epub 2020 Oct 21. PMID: 33081526.

Teaching Points

After a simulation scenario, feel free to ask 1 or 2 a quick debrief questions, such as “What was hard about that?,” but do not spend too much time in reflection as the focus after simulations should primarily be on these teaching points and practical skills. There are 4 universal teaching points to include between simulation cycles. Three mini-lectures are typically given after the first scenario run-through:

- Rapid Assessment and Calling for More Help

- Basic Airway and Ventilation Skills

- Timing and Choreography of Pad Placement and Defibrillation

The fourth mini-lecture is typically given after the 2^nd^ or 3^rd^ scenario run-through:

- Optimizing Environment

**Rapid Assessment and Calling for More Help:**

*Intro:* Calling for help early is critical to escalate the care provided to patients. You need help whenever you do not have the resources to provide definitive care for the patient, or when you may need more resources very soon. For example, if you walk into a room and the patient is unresponsive (or in respiratory distress, or shock, or seizing). There is no need to perform more evaluation or begin resuscitation prior to at least calling down the hall for more help.

*Actions:* Point out that to get the overhead page, someone needs to call 7-9999. Show where to find room number in the room. (You should adjust this teaching point to match your institution’s emergency alert system).

**Basic Airway and Ventilation Skills:**

*Intro:* Most pediatric arrests are respiratory in nature. Knowing how to adequately ventilate a patient during resuscitation is an essential skill.

On the acute care units, we have access to self-inflating bags, which do not require pressurized air/oxygen. In contrast to a flow-inflating bag, you cannot give CPAP or blow-by oxygen with a self-inflating bag. The rate of respirations should be 15:2 in a patient < 8 years old and 30:2 in a patient > 8 years old.

*Action:* Walk learners through ventilation steps (*with demonstration depending on level of learners*):

1. Proper positioning – In the absence of neck injury, tilt the forehead back and lift the chin

2. Tight seal – Use the “E-C clamp,” which is the letters E and C formed by the fingers and thumb over the mask

3. Ventilate – Squeeze the bag over one second until the chest rises.

**Timing and choreography of pad placement and defibrillation:**

*Intro:* Minimizing interruptions is one of the components of high-quality CPR. The defibrillator is more important to place than other monitors during a code.

*Action:* Show learners how to choreograph placement of pads and backboard. Show learners how to turn on defibrillator and plug in leads. Depending on level of learners, show how to charge and adjust defibrillation dose (2J/kg and later 4J/kg; round up).

**Optimizing Environment:**

*Intro:* The room set-up and situation on the acute care floor is often sub-optimal for resuscitation. In order to assess and resuscitate a patient, responders need to optimize the setting by turning on lights, removing blankets or additional items from the bed, re-positioning the bed, etc.

*Action:* Show trainees where various resuscitations supplemental tools are located in the acute care room (e.g., stool), how to lower crib rails, move bed or head of bed.
