# Supplementary material for: First 3 Minutes: A Rapid Cycle Deliberate Practice Pediatric Resuscitation Simulation for Multidisciplinary Staff
Source: MedEdPORTAL. 2025 Jun 6;21:11529. doi: 10.15766/mep_2374-8265.11529 (PMC12141546; doi:10.15766/mep_2374-8265.11529)
Supplement: Supplementary file 1 — First 3 Minutes Facilitator Guide.docxSimulation Scenario with Critical Action Points.docxFacilitator Scripts and Teaching Points.docxVisual Aid with Simulation Objectives.docxPrintable Team Role Cards.docxPreparticipation Survey and CPR Test.docxPostparticipation Survey and CPR Test.docxKey Take-Home Points for Learners.docx [file mep_2374-8265.11529-s001.zip › E. Printable Team Role Cards.docx]

**Appendix E: Printable Team Role Cards**

In this appendix, you will find participant role cards that can be printed and handed to participants so that when their roles are assigned, they have a visual reminder of what they should be doing in the simulation.

**Team Roles:**

**1^st^ responder:** focus on initial rapid assessment (Appearance, Breathing, and Color), calling for help, and then turning back to pulse & perfusion. Then if not compressing chest, aid with other aspects (physical examination, monitors, medications).

**2^nd^ responder:** inquire if code has been called, focus on airway, oxygen, ventilation.

**3^rd^ responder:** obtain code cart, focus on applying defibrillation pads, and placing backboard if needed. Then check access & aid with medications.

**Team Roles:**

**1^st^ responder:** focus on initial rapid assessment (Appearance, Breathing, and Color), calling for help, and then turning back to pulse & perfusion. Then if not compressing chest, aid with other aspects (physical examination, monitors, medications).

**2^nd^ responder:** inquire if code has been called, focus on airway, oxygen, ventilation.

**3^rd^ responder:** obtain code cart, focus on applying defibrillation pads, and placing backboard if needed. Then check access & aid with medications.

**Team Roles:**

**1^st^ responder:** focus on initial rapid assessment (Appearance, Breathing, and Color), calling for help, and then turning back to pulse & perfusion. Then if not compressing chest, aid with other aspects (physical examination, monitors, medications).

**2^nd^ responder:** inquire if code has been called, focus on airway, oxygen, ventilation.

**3^rd^ responder:** obtain code cart, focus on applying defibrillation pads, and placing backboard if needed. Then check access & aid with medications.
