# Supplementary material for: First 3 Minutes: A Rapid Cycle Deliberate Practice Pediatric Resuscitation Simulation for Multidisciplinary Staff
Source: MedEdPORTAL. 2025 Jun 6;21:11529. doi: 10.15766/mep_2374-8265.11529 (PMC12141546; doi:10.15766/mep_2374-8265.11529)
Supplement: Supplementary file 1 — First 3 Minutes Facilitator Guide.docxSimulation Scenario with Critical Action Points.docxFacilitator Scripts and Teaching Points.docxVisual Aid with Simulation Objectives.docxPrintable Team Role Cards.docxPreparticipation Survey and CPR Test.docxPostparticipation Survey and CPR Test.docxKey Take-Home Points for Learners.docx [file mep_2374-8265.11529-s001.zip › A. First 3 Minutes Facilitator Guide.docx]

Appendix A: First Three Minutes Facilitator Guide

This appendix will guide you through the steps and content required to facilitate a First Three Minutes simulation.

**List of Appendices:**

A. First Three Minutes Facilitator Guide.docx

B. Simulation Scenario with Critical Action Points.docx

C. Facilitator Scripts and Teaching Points.docx

D. Visual Aid with Simulation Objectives.docx

E. Printable Team Role Cards.docx

F. Pre-Participation Survey and CPR Test.docx

G. Post-Participation Survey and CPR Test.docx

H. Key Take Home Points for Learners.docx

**Simulation Objective:**

The goal of this project is to provide brief, focused simulation trainings to increase competency and confidence for acute care providers as first responders to in-hospital respiratory and cardiac emergencies. This simulation focuses on the hands-on and practical skills required to initiate CPR and prepare for the arrival of the Code Blue team. As opposed to the typical debriefing and reflective learning experienced in many simulations, this simulation is geared towards rapid cycle deliberate practice, a technique that involves learners repeatedly performing a simulation with micro-debriefs interjected by the instructor. The specific simulation goals are to 1) identify an unresponsive patient and call for help appropriately, 2) increase confidence in addressing airway, breathing, and circulation in the first 3 minutes of a code situation, and 3) recall high-quality CPR principles.

**Simulation Setting and Required Materials:**

This simulation takes place in an acute care floor hospital room. It can be run in other settings (i.e., conference room), but it is ideally run in a room where the instructor can point out specific aspects of the patient room that are important for code situations (e.g., code button, emergency airway supplies box, room number located on the door frame).

| **Required Materials:** |
| --- |
| Low tech CPR doll (high tech could also be used if we have the resources) |
| Training code cart with backboard, defibrillator, defibrillator pads |
| Pediatric mask with inflatable cushion |
| Pediatric self-inflating ventilation bag |
| Stool (for compressions) |

**Simulation participants:**

This simulation is designed for a group of 3 learners. More learners may participate through observation and cycling through the scenario, but it is primarily set up for 3 participants. Learners can be healthcare staff who have training in PALS, PEARS, or BLS.

**Session Flow:**

This training session should last about 30 minutes, with a pre- and post-participation survey for each learner not included in that time frame (see approximate timing below). The short simulation case will be repeated 3 times, with each participant cycling through each role.

**Simulation Scenario and Critical Action Points:**

The simulation scenario and critical action points are found in Appendix B. After the Simulation Pre-Briefing (see below), the first simulation should be conducted without any stoppages, so that the learners can feel what it would be like to respond to a code. There is typically a debrief after the 1^st^ simulation during which the participants receive 3 of the 4 universal teaching points and have any questions answered (see Debriefing and Teaching Points below). You can then proceed with the 2^nd^ and 3^rd^ simulations, which should be stopped at critical points to allow participants to receive feedback on the learning objectives and try again.

For each simulation, decide in advance who will be 1^st^, 2^nd^, and 3^rd^ responder. The first responder should focus on initial rapid assessment, calling for help, and then turning back to pulse and perfusion. The second responder should focus on airway, oxygen, ventilation. The third responder should focus on applying defibrillation pads and placing backboard if needed. When the parent calls for help (voiced by the facilitator), the first responder can enter the room immediately. They should start their assessment and call for extra help when needed. At this time, one of their colleagues will come in immediately while the remaining team members will go and retrieve the code cart. They should wait at least 15 seconds before entering to mimic the normal delay of getting the code cart.

In the simulation scenario, there are suggested hard and soft stopping points for the 2^nd^ and 3^rd^ simulations. These are points at which the RCDP simulation should be stopped if the participants do not perform the expected actions or do not demonstrate appropriate usage of equipment. They should receive feedback about the actions and then be allowed to try again. After feedback, you can restart the case from the beginning or just before the stoppage.

**Simulation Pre-Briefing:**

Suggested scripting for the Pre-Simulation Briefing can be found in Appendix C. The Pre-Simulation Briefing should include creating a safe learning environment, an orientation to the simulation, an orientation to the room and mannequin, a review of high-quality CPR principles, and an orientation to rapid cycle deliberate practice.

**Debriefing and Teaching Points**

To debrief after the first simulation scenario, feel free to ask 1 or 2 a quick debrief questions, such as “What was hard about that?” but do not spend too much time in reflection as the focus after simulations should primarily be on the teaching points and practical skills. There are 4 universal teaching points to include during simulation cycles. Suggested scripting for the teaching points can be found in Appendix C. Three mini-lectures are typically given after the first scenario run-through:

- Rapid Assessment and Calling for More Help

- Basic Airway and Ventilation Skills

- Timing and Choreography of Pad Placement and Defibrillation

The fourth mini-lecture (“Optimizing Environment”) is typically given after the 2^nd^ simulation.

**Visual Aid with Simulation Objectives**

In Appendix D, you will find a visual aid that you can print and post in the simulation room to help learners identify their specific learning objectives.

**Participant Role Cards**

In Appendix E, you will find participant role cards that can be printed and handed to participants so that when their roles are assigned, they have a visual reminder of what they should be doing in the simulation.

**Pre- and Post-Participation Surveys**

In Appendix F and G, you will find the pre- and post-participation surveys, respectively, used at our institution.

**Key Take Home Points**

In Appendix H, you will find a Key Take Home Points sheet that should be printed and distributed to participants at the end of the session.

**Approximate Session Timing**

Prior to start time: Pre-participation survey

Minutes 0-5: Introductions, Orientation to Simulation, Review of High-Quality CPR Components

Minutes 5-10: 1^st^ simulation

Minutes 10-20: Lessons on Calling for Help, Basic Airway Skills, Timing and Choreography of Defibrillator Pad Placement

Minutes 20-28: 2^nd^ & 3^rd^ simulation with lesson on Optimizing Environment

Minute 28-30: Final Debrief and distribute Key Take Home Points

After session: Post-participation survey
