# Supplementary material for: First 3 Minutes: A Rapid Cycle Deliberate Practice Pediatric Resuscitation Simulation for Multidisciplinary Staff
Source: MedEdPORTAL. 2025 Jun 6;21:11529. doi: 10.15766/mep_2374-8265.11529 (PMC12141546; doi:10.15766/mep_2374-8265.11529)
Supplement: Supplementary file 1 — First 3 Minutes Facilitator Guide.docxSimulation Scenario with Critical Action Points.docxFacilitator Scripts and Teaching Points.docxVisual Aid with Simulation Objectives.docxPrintable Team Role Cards.docxPreparticipation Survey and CPR Test.docxPostparticipation Survey and CPR Test.docxKey Take-Home Points for Learners.docx [file mep_2374-8265.11529-s001.zip › F. Preparticipation Survey and CPR Test.docx]

**Appendix F: Pre-participation survey**

1. SCH Badge Number (for linking pre- and post-surveys, not for identification or any other use)

________________

2. Job title:

1. Resident/Fellow
2. Nurse
3. Respiratory Therapist
4. Attending Physician
5. APP
6. Other (fill in)

2. b. PGY level (for residents/fellows)

1. PGY-1
2. PGY-2
3. PGY-3
4. PGY-4 or greater

2. c. I have been practicing for: (for non-residents/fellows)

1. 1-3 years
2. 4-6 years
3. 6-9 years
4. 10 years or greater

3. Regarding resuscitation training, I have attend PALS/PEARS/BLs in the last:

1. 6 months
2. 12 months
3. 24 months

4. Number of code events requiring CPR that I have participated in during the last 3 years:

1. 0
2. 1
3. 2
4. 3
5. 4
6. 5 or more

5. I am confident I have the skills and knowledge to provide high-quality CPR. (pick one)

Strongly disagree Disagree Neither agree nor disagree Agree Strongly agree

6. I am confident I have the skills and knowledge to assess and emergently manage airway, breathing, and circulation in the first 3 minutes of a code situation. (pick one)

Strongly disagree Disagree Neither agree nor disagree Agree Strongly agree

6. What would make you feel more confident in responding to codes?

**Principles of High Quality CPR Pre-Test**

1. What is the compression to breath ratio when performing CPR on an infant or child with two providers?

1. 30:2
2. 25:2
3. 20:2
4. 15:2

2. What is the depth of compressions in pediatric CPR?

A. At least 1/4 the depth of the child’s chest

B. At least 1/3 the depth of the child’s chest

C. At least 1/2 the depth of the child’s chest

D. At least 2/3 the depth of the child’s chest

3. What is the rate of compressions in pediatric CPR?

A. 60-80 per minute

B. 80-100 per minute

C. 100-120 per minute

D. 120-140 per minute

4. Which of these is NOT a necessary component of high quality CPR?

A. Full chest recoil between compressions

B. Minimal interruptions during chest compressions

C. Having a back board in place

D. Avoiding excessive ventilation
