# Supplementary material for: First 3 Minutes: A Rapid Cycle Deliberate Practice Pediatric Resuscitation Simulation for Multidisciplinary Staff
Source: MedEdPORTAL. 2025 Jun 6;21:11529. doi: 10.15766/mep_2374-8265.11529 (PMC12141546; doi:10.15766/mep_2374-8265.11529)
Supplement: Supplementary file 1 — First 3 Minutes Facilitator Guide.docxSimulation Scenario with Critical Action Points.docxFacilitator Scripts and Teaching Points.docxVisual Aid with Simulation Objectives.docxPrintable Team Role Cards.docxPreparticipation Survey and CPR Test.docxPostparticipation Survey and CPR Test.docxKey Take-Home Points for Learners.docx [file mep_2374-8265.11529-s001.zip › G. Postparticipation Survey and CPR Test.docx]

**Appendix G: Post-participation survey**

1. How helpful did you find this training session? (pick one)

Not at all helpful Not so helpful Somewhat helpful Very helpful Extremely helpful

2. After this training, I am confident I have the skills and knowledge to provide high-quality CPR. (pick one)

Strongly disagree Disagree Neutral Agree Strongly agree

2. After this training, I am confident I have the skills and knowledge to assess and emergently manage airway, breathing, and circulation in the first 3 minutes of a code situation. (circle one)

Strongly disagree Disagree Neutral Agree Strongly agree

3. How often would it be helpful to repeat this training session (e.g., every month, each wards rotation, once every 6 months, once a year, not helpful to repeat, etc.)

4. What is one learning point or skill you will take away from this simulation?

5. How could this training session be improved in the future?

**Principles of High Quality CPR Post-Test**

1. What is the compression to breath ratio when performing CPR on an infant or child with two providers?

1. 30:2
2. 25:2
3. 20:2
4. 15:2

2. What is the depth of compressions in pediatric CPR?

A. At least 1/4 the depth of the child’s chest

B. At least 1/3 the depth of the child’s chest

C. At least 1/2 the depth of the child’s chest

D. At least 2/3 the depth of the child’s chest

3. What is the rate of compressions in pediatric CPR?

A. 60-80 per minute

B. 80-100 per minute

C. 100-120 per minute

D. 120-140 per minute

4. Which of these is NOT a necessary component of high quality CPR?

A. Full chest recoil between compressions

B. Minimal interruptions during chest compressions

C. Having a back board in place

D. Avoiding excessive ventilation
