# Supplementary material for: First 3 Minutes: A Rapid Cycle Deliberate Practice Pediatric Resuscitation Simulation for Multidisciplinary Staff
Source: MedEdPORTAL. 2025 Jun 6;21:11529. doi: 10.15766/mep_2374-8265.11529 (PMC12141546; doi:10.15766/mep_2374-8265.11529)
Supplement: Supplementary file 1 — First 3 Minutes Facilitator Guide.docxSimulation Scenario with Critical Action Points.docxFacilitator Scripts and Teaching Points.docxVisual Aid with Simulation Objectives.docxPrintable Team Role Cards.docxPreparticipation Survey and CPR Test.docxPostparticipation Survey and CPR Test.docxKey Take-Home Points for Learners.docx [file mep_2374-8265.11529-s001.zip › H. Key Take-Home Points for Learners.docx]

**Appendix H: Key Take Home Points for Learners**

In this appendix, you will find a Key Take Home Points sheet that can be printed and distributed to participants at the end of the session.

**First 3 Minutes Learning Points:**

**Rapid Assessment and Calling for More Help:**

Calling for help early is critical to escalate the care provided to patients. You need help whenever you do not have the resources to provide definitive care for the patient, or when you may need more resources very soon.

Within our hospital, in order to get the overhead page, someone needs to call 7-9999 and tell the operator the room number.

**High quality CPR:**

Components of high quality CPR (based on 2020 AHA guidelines^1^):

- providing adequate chest compression rate (≈100–120/min)
- providing adequate chest compression depth (depress the chest at least 1/3 the A-P diameter of the chest, approximately 1.5 inches (4 cm) in infants to 2 inches (5 cm) in children.
- minimizing interruptions in CPR
- allowing full chest recoil between compressions
- avoiding excessive ventilation

**Basic Airway and Ventilation Skills:**

Most pediatric codes are respiratory in nature. In 2-rescuer CPR without an advanced airway, the rate of respirations should be 15:2 in a patient < 8 years old and 30:2 in a patient > 8 years old.

Technique:

1. Position patient – in the absence of neck injury, tilt the forehead back and lift the chin
2. Obtain tight seal – use “E-C clamp” with fingers over the mask
3. Ventilate – squeeze bag over 1 second until the chest rises

**Timing and choreography of pad placement and defibrillation:**

The defibrillator pads are more important than other monitors to place during a code. Use pediatric pads for patients less eight years old or less than 25 kg (55 lbs). When setting up the defibrillator, instruct the compressor to continue compressions while you get prepared. Coordinate flipping the patient, placing back pad, and placing backboard at the same time at the end of a cycle of compressions. The starting dose for defibrillation is 2 J/kg.

**Optimizing Environment:**The room set-up and situation on the acute care floor is often sub-optimal for resuscitation. In order to assess and resuscitate a patient, responders need to optimize the setting by turning on lights, removing blankets or additional items from the bed, re-positioning the bed, etc.
